# Historical Refugia and Isolation by Distance of the Mud Snail, *Bullacta exarata* (Philippi, 1849) in the Northwestern Pacific Ocean

**DOI:** 10.3389/fgene.2018.00486

**Published:** 2018-10-22

**Authors:** Lu-Ye Shi, Jia Li, Shu-Qing Wu, Jie Han

**Affiliations:** Ministry of Education Key Laboratory for Biodiversity Science and Ecological Engineering, College of Life Sciences, Beijing Normal University, Beijing, China

**Keywords:** *Bullacta exarata*, historical refugia, isolation by distance, the Northwestern Pacific, phylogeography

## Abstract

Many phylogeographic studies on marine organisms in the Northwestern Pacific have supported for the biogeographic hypotheses that isolation in the marginal seas of this region during the Pleistocene glaciation lower sea level led to population genetic divergence, and thus population expansion was a common phenomenon when the sea level rebounded. However, most of these studies were based on maternally inherited mitochondrial DNA markers with limited sample sites and therefore, were unable to reveal detailed pictures encompassing paternal line information covering of the entire range. In this study, we used the mitochondrial cytochrome c oxidase subunit I (COI) and nine nuclear microsatellite loci to investigate the phylogeography of the mud snail, *Bullacta exarata* (Philippi, 1849), a species endemic to the Northwestern Pacific. We sampled 14 natural populations spanning across 3800 km of the Chinese coastline, essentially covering most of the species distribution range. COI analysis identified a total of 149 haplotypes separated into two distinct groups with nine mutation steps, revealing a prominent phylogeographic structure. Nuclear microsatellite data also demonstrated a similar but weaker genetic structure. The estimated time to the most recent common ancestor between the two COI haplogroups is at ∼0.89 Ma, indicating that *B. exarata* populations survived the Pleistocene glaciation in the Sea of Japan and the Okinawa Trough, two marginal seas around the species range. The consistent significant patterns of isolation by distance of both COI and microsatellites suggests that limited mobility of adults and short planktonic stage of larvae may have played an important role in promoting or maintaining the genetic differentiation of *B. exarata*. Results from population demographic analyses support population expansion late in the Pleistocene era.

## Introduction

Many phylogeographic studies on marine organisms in the Northwestern Pacific have supported the biogeographic hypothesis of the vicariant events (Ni et al., 2014). And this is particularly associated with the unique geography of this area, presenting a series of deeper basins, or marginal seas between the mainland and the ocean (Wang, 1999). During the Pleistocene era when climate changed drastically, this area experienced repeated sea level fluctuations and interchanges between glaciation and deglaciation. When the glaciers advanced, the sea level in the East China Sea dropped to a depth which is about 130–150 m deeper than that of present day. The Bohai Sea, Yellow Sea, and the shelf of the East China Sea were exposed as the coastline migrated about 1200 km seawards (Wang, 1999; Shen et al., 2011; Ni et al., 2014). Many marine species thus lost their suitable habitats and went extinct, while others survived in the marginal seas acting as the glaciation refugia (Shen et al., 2011; Ni et al., 2014). Evidence of these events can be found in the phylogeographical structure of marine species, and the number of divergent lineages in a species is often assumed to be related to the number of refugia (Barber et al., 2000; Lourie et al., 2005; Liu et al., 2007). During the deglaciation and post glaciation periods, the sea level rebounded and the isolated populations potentially expanded to colonize the recovered habitats rapidly (Ni et al., 2012). Species, such as the sea bass, turban shells, mitten crabs and algae in the genus *Sargassum* provide evidence of allopatric divergence and expansion closely associated with the sea level changes in the Northwestern Pacific during the Pleistocene era (Kojima et al., 1997; Liu et al., 2007; Cheang et al., 2008, 2010; Xu et al., 2009). However, most of these studies were based on maternally inherited mitochondrial DNA markers with limited sample sites and were therefore unable to reveal detailed pictures including paternal line information covering the whole range (Ni et al., 2014).

In addition to the impactful palaeoclimate events, the dispersion of planktonic larvae is another important factor for the phylogeographic structure of marine organisms (Bertness and Gaines, 1993; Tsang et al., 2008). It is generally known that the longer the stage of planktonic larvae, the longer the distance of migration and dispersion. For example, the abalone *Haliotis rubra*, with only a 6-day pelagic larval phase, has a dispersal distance of less than 15 km, the bamboo clam *Ensis directus*, which has a 16-day pelagic larval phase, can disperse about 111 km away, and the brown mussel *Perna perna*, which has a 15 to 20-day pelagic larval phase, can disperse about 235 km from its spawning sites (Shanks et al., 2003). According to theory of population genetics, a longer planktonic larval stage would result in increased gene flow among geographically distant populations, and consequently levels of their genetic differentiation would decrease. For instance, a solitary coral species with a brief planktonic larval stage showed a stronger population genetic structure than that with a longer larval stage (Hellberg, 1996).

The mud snail *Bullacta exarata* (Philippi, 1849) belongs to the superfamily Haminoeoidea (Mollusca: Gastropod: Cephalaspidea) and is an endemic species of the Northwestern Pacific, encompassing China from the East China Sea northwards, the Korean Peninsula, and the coast of Japan (Ye and Lu, 2001; Malaquias, 2010). *B. exarata* is a simultaneous hermaphrodite organism with euryhaline distribution, and the adults dwell on the intertidal mudflat with limited mobility. During breeding season, which occurs from March to November, sexually mature adults can reproduce multiple times and produce more than 6000 fertilized eggs each time. After spawning, developed larvae experience a 4–10 days pelagic life before converting to creeping stage (You et al., 2003). The pelagic larvae may be transferred by ocean currents, causing dispersal of this species. In this study, we use the mitochondrial cytochrome c oxidase subunit I (COI) gene and nine nuclear microsatellite loci to investigate the phylogeographic structure and demographic history of *B. exarata* in the Northwestern Pacific Ocean. We aim to test (1) whether the Pleistocene marginal sea refugia have left any genetic imprint on *B. exarata*, (2) whether the short planktonic larval stage have promoted the genetic structure of this species. By addressing these issues, we hope to gain a better understanding of the genetic diversity distribution and the evolutionary mechanisms of marine organisms along the Northwestern Pacific Ocean.

## Materials and Methods

### Population Sampling and DNA Extraction

**FIGURE 1:**
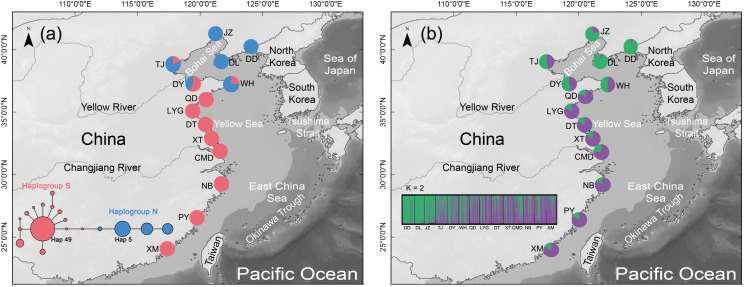
**(a)** Distribution of the two COI haplogroups in the mud snail, *Bullacta exarata*. Populations are labeled with IDs as shown in Supplementary Table S1. Light gray shading along the shoreline depicts 120 m continental depth. Sea level was at or below this depth during glacial periods of the Pleistocene. The medium-joining network of haplotypes which only displays torso for clarity, the whole MJ network of 149 haplotypes was shown in the Supplementary Figure S1. Circle sizes are proportional to haplotype frequency, and the smallest circle represents one haplotype. Each branch represents one mutational difference unless labeled with number indicating the number of mutations. **(b)** Geographic distribution of *B. exarata* populations and their color-coded grouping at the most likely *K* = 2.

We collected 432 adult individuals from 14 natural populations spanning 3800 km of coastline distance along the coast of China and covering almost the entire distribution range of *B. exarata* (Figure 1 and Supplementary Table S1). All individuals were identified according to the morphological characters, and then were preserved in absolute ethanol. Total genomic DNA was extracted from foot tissue using a protocol as described previously (Marko, 2002; Supplementary Table S5).

### The COI Gene Sequencing

The COI gene was amplified using polymerase chain reaction (PCR) with primers HCO-2198 and LCO-1490 from Folmer et al. (1994). PCR was performed in a total volume of 10 μL including approximately 40 ng of template DNA, 0.16 μM of each primer, and 5 μL of 2 × EasyTaq SuperMix (Transgen Biotech). The thermal cycle conditions were template denaturation at 95°C for 5 min, followed by 35 cycles of denaturing at 95°C for 45 s, annealing at 45.7°C for 45 s and extension at 72°C for 60 s, and then a final extension at 72°C for 10 min. PCR products were purified using the E.Z.N.A.^®^ Gel Extraction Kit (Omega Bio-Tek Inc., GA, United States). Bi-directional sequencing was conducted using the PCR primers and the BigDye^®^ Terminator Cycle Sequencing Kit (v3.1) on an ABI PRISM^®^ 3730 Genetic Analyzer (both from Applied Biosystems, Foster City, CA, United States).

### Microsatellite Genotyping

Genotypes of DNA samples were scored using nine microsatellite loci developed for *B. exarata* (Du et al., 2010; Wang et al., 2010; Supplementary Table S2). PCR cocktail and thermal cycle conditions were the same as the COI gene except for the annealing temperatures (see Supplementary Table S2). Loci of three randomly selected individuals were re-amplified and scored to act as positive control for genotyping methods. GeneScan^TM^-500LIZ^TM^ (Applied Biosystems) was used to determine the allele size. Fragment sizes were obtained on an Applied Biosystems 3730 and scored using GeneMapper version 3.7 (Applied Biosystems). About 30% samples were reamplified, and all samples were scored twice independently to check for genotyping consistency.

### The COI Gene Sequence Analyses

#### Genetic Diversity

Forward and reverse sequences of the COI gene were assembled and edited with SeqMan Pro program (Lasergene version 7.1, DNASTAR, Madison, WI, United States) (Swindell and Plasterer, 1997), and were then aligned using CLUSTAL_X (Thompson et al., 1997). The number of haplotypes (*Nh*), haplotype diversity (*Hd*), and nucleotide diversity (π) were calculated with DnaSP v.5.0 (Librado and Rozas, 2009).

#### Phylogenetic Analysis

Phylogenetic relationships and frequencies of the COI gene haplotypes were determined by constructing a median-joining network using Network v4.6 (Bandelt et al., 1999). The time to most recent common ancestor (*t_MRCA_*) was estimated using BEAST v1.8.2 (Doonan et al., 2012). The prior settings were the General Time Reversible (GTR) model for nucleotide substitution with four gamma categories, the uncorrelated lognormal relaxed molecular clock model with the mean of the branch rates constrained to 2.4% per million years (Myr^−1^) as estimated for the COI gene of two marine teguline snails separated by the Isthmus of Panama (Hellberg and Vacquier, 1999), and the Yule process of speciation with a random starting tree. Three independent runs were executed, with 10 million Markov Chain Monte Carlo (MCMC) generations, sampling every 1000 generations. The convergence and mixing of the MCMC chains were assessed using Tracer v1.6 (Rambaut and Drummond, 2007).

#### Population Genetic Structure and Isolation by Distance (IBD) Analysis

Population genetic structure was detected with pairwise *F_ST_* between populations and analysis of molecular variance (AMOVA) with Arlequin 3.0 (Excoffier et al., 2005). To test IBD in *B. exarata* populations, the Mantel test was performed with IBD web service^1^ (Bohonak, 2002; Jensen et al., 2005). The correlation between the geographical distance and genetic distance was analyzed. The genetic distances were expressed as *F_ST_*/(1 – *F_ST_*). The geographical distances between populations were represented by the coastline distance calculated using ArcGIS (ESRI, Redlands, CA, United States).

#### Historical Demography of Different Haplotype Groups

To assess historical demography of haplogroups, we used three methods: neutrality test, mismatch distribution analysis and Bayesian skyline plot (BSP). In the neutrality test, the values of Tajima (1989) and Fu (1997) were calculated. Negative values significantly deviating from the null distributions can be interpreted as signatures of population expansion. The mismatch distribution is multimodal if samples drawn from a population at demographic equilibrium, and it is unimodal if samples drawn from a population with a recent demographic or range expansion (Rogers and Harpending, 1992; Ray et al., 2003). The time since expansion (t) was estimated using τ in the equation *t* = τ/2 μ*k*, where τ is calculated in the mismatch distribution analysis, μ is the mutation rate, and *k* is the length of sequence. Both mismatch analysis and neutrality test were performed in Arlequin 3.0. BSP (Drummond et al., 2005) was generated to model the historical changes in effective population size using the coalescent approach with BEAST v1.8.2 and visualized with TRACER v1.6.

### Microsatellite Data Analyses

#### Genetic Diversity

Possible genotyping errors due to null allele, stuttering or large allele drop out within each locus were assessed with Micro-Checker version 2.2.3 (Van Oosterhout et al., 2004). The departure from Hardy-Weinberg Equilibrium (*HWE*) at each locus and linkage disequilibrium between all pairs of loci were tested using MCMC approach implemented in GENEPOP version 4.0 (Rousset, 2008). All multiple comparisons were adjusted with Bonferroni corrections (Rice, 1989). Genetic diversity indices including the number of alleles (*Na*), the observed (*Ho*), and expected (*He*) heterozygosities and the value of pairwise *F_ST_* between populations were calculated using Arlequin 3.0.

#### Population Structure Analysis

We used a Bayesian clustering algorithm implemented in STRUCTURE v.2.3.4 (Pritchard et al., 2000) to infer population structure and explore the assignment of individuals and populations to specific genetic clusters. The analysis involved an admixture model assuming correlations among allele frequencies across groups. The model was tested for each assumed *K* cluster of 2 ≤*K* ≤ 14 with 20 independent simulations running 200000 MCMC steps, with the initial 50000 steps as burn-in. The average permutated individual Q-matrices throughout the 20 simulations for the estimated number of clusters, i.e., *K* value, were obtained using greedy algorithm in CLUMPP v.1.1.2 (Jakobsson and Rosenberg, 2007), and the simulated results were visualized in DISTRUCT (Rosenberg, 2004). To estimate the most optimal *K*, we used three approaches. First, we used the best log-likelihood score [lnPr(*D*)], resulting in the highest percentage of membership coefficient (q) to each cluster (Pritchard et al., 2000). Second, Δ*K* = mean[| L”(*K*)| ]/SD[L(*K*)], i.e., rate of change in the log probability of data between successive *K* values, was plotted against *K* and the optimal *K* identified by the largest change in log-likelihood [L(*K*)] values between *K* values (Evanno et al., 2005). Third, we adopted the suggestion from Pritchard et al. (2000) that for real-world data in which identifying the correct *K* is not always straightforward, the most optimal *K* should be the one that reveals a biologically meaningful genetic structure. The first and second approaches were carried out using the website program STRUCTURE HARVESTER^2^ (Earl and Vonholdt, 2012).

The analysis of IBD and AMOVA were also performed using the same methods as in the COI gene data analyses.

## Results

### Genetic Diversity

The aligned COI gene sequences were 631 bp in length, including 136 variable sites with 26 transversions and 110 transitions. Totally 149 haplotypes were defined (All data used in the study has been uploaded to GenBank, and can be downloaded by accession numbers MH827086–MH827517), among which haplotype 49 (Hap49) was the most common and was mainly present in the southern populations from WH southward, while Hap5 was the second most common haplotype and was mainly existed in the northern populations from QD northward (Figure 1a, Supplementary Table S1, and Supplementary Figure S1). The mean values of *Nh*, *Hd*, and π across 14 populations were 13.79, 0.7633, and 0.0236, respectively (Table 1).

**Table 1:** Population ID and genetic diversity indices of *B. exarata* based on the COI gene and nine nuclear microsatellite loci: *Nh*, number of haplotypes; *Hd*, haplotype diversity; π, nucleotide diversity; *H*e, expected heterozygosity; *H*o, observed heterozygosity; *Na*, number of alleles.

ID	COI			Microsatellites		
	*Nh*	*Hd*	π	*He*	*Ho*	*Na*
DD	14	0.6895	0.0201	0.7983	0.6121	13.44
DL	20	0.9254	0.0330	0.8233	0.6242	13.56
JZ	15	0.9355	0.0308	0.8516	0.6722	14.67
TJ	11	0.8930	0.0562	0.8480	0.6315	14.00
DY	12	0.7908	0.0578	0.8650	0.6358	16.56
WH	19	0.9264	0.0326	0.8601	0.6654	12.22
QD	9	0.5062	0.0081	0.8021	0.5542	11.89
LYG	13	0.6851	0.0094	0.7616	0.6402	11.00
DT	13	0.6851	0.0088	0.8330	0.6721	12.33
XT	17	0.8184	0.0207	0.8266	0.6418	12.33
CMD	10	0.7839	0.0127	0.7936	0.7385	12.22
NB	14	0.5609	0.0108	0.8484	0.6489	13.67
PY	11	0.6995	0.0164	0.8135	0.7261	11.78
XM	15	0.7862	0.0124	0.8630	0.7077	13.11
Mean	13.79	0.7633	0.0236	0.8278	0.6551	13.06

For the microsatellite data, no evidence for null allele or stuttering or large allele drop out was present at the nine loci within each sampling sites or when all samples were pooled (*P* > 0.05). None of the loci showed significant deviation from *HWE*, and no linkage equilibrium was found between any pairs of loci after Bonferroni corrections. Total *Na*, *He*, and *Ho* across 14 populations were 13.06, 0.8278, and 0.6551, respectively (Table 1).

### Population Structure

Median-joining network revealed the 149 COI haplotypes clustered into two haplogroups with nine mutation steps between them, displaying a prominent geographic cline. Haplogroup S mainly distributed in the southern populations from WH southward, while haplogroup N dominated the northern populations from QD northward (Figure 1a and Supplementary Figure S1). The estimated *t_MRCA_* for the two haplogroups was 0.8878 Ma (95% HPD: 0.4851–1.4225), a time falling into the Pleistocene era (2.58–0.01 Ma) (Meyers and Hinnov, 2010). The results of AMOVA further supported genetic differentiation between the southern and northern populations with a very significant variation of 72.18% (Table 2).

**Table 2:** The results of AMOVA for the south (WH southward) and north (QD northward) population groups of *B. exarata* based on the COI gene (a) and microsatellite loci (b).

Source of variation	Percentage variation a/b	*P*-value a/b
Among groups	72.18/1.91	<0.001/<0.001
Among populations within groups	5.50/1.82	<0.001/<0.001
Within populations	22.32/96.19	<0.001/<0.001

To select the most suitable *K* using microsatellite data set in the structure analysis, the best log-likelihood score approach “LnP(*D*)” was first applied, but no distinct plateau of the estimates of LnP(*D*) was observed (Supplementary Figure S2). We then plotted the alternative statistic Δ(K against K (Supplementary Figure S2). The curve revealed a prominent peak of Δ(K value at K ( 2, indicating two distinct groups. These two groups had a geographic distribution pattern the same to those of the two COI gene haplogroups, with one group is mainly distributed QD southward and another WH northward (Figure 1b). AMOVA showed a small but still significant variation (1.91%) between the two population groups as analyzed using the COI gene (Table 2).

**FIGURE 2:**
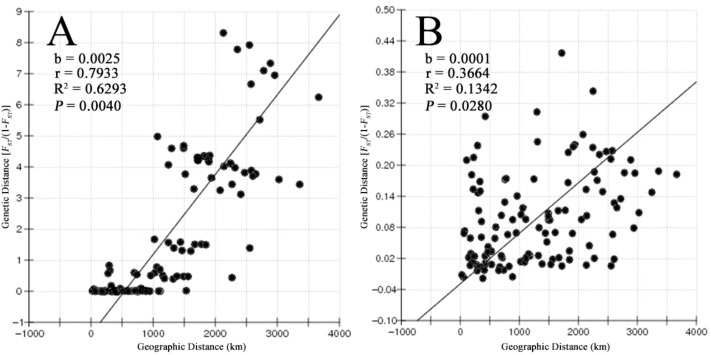
The result of isolation by distance analysis based on the mitochondrial COI gene **(A)** and nine microsatellites **(B)**.

Most of the pairwise differences between populations revealed by pairwise *F_ST_* values based on the COI gene and microsatellite data were significant after the Bonferroni correction. The values between adjacent populations were comparatively lower (Supplementary Table S3). The results of the Mantel test showed a strong positive correlation between geographic distance and genetic distance (*r* = 0.7933, *P* = 0.004 for COI and *r* = 0.3664, *P* = 0.028 for microsatellites), supporting a pattern of IBD in *B. exarata* (Figures 2A,B).

### Historical Demography of Different Haplogroups

**FIGURE 3:**
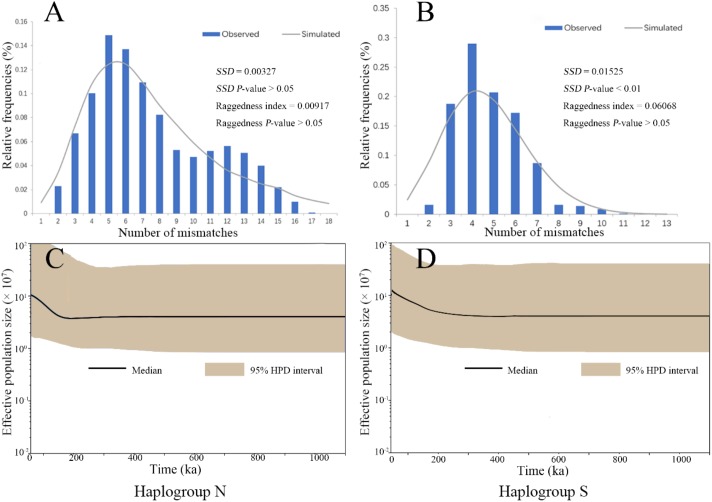
The analysis of mismatch distribution **(A,B)** and Bayesian skyline plot **(C,D)** for the two COI haplogroup populations of *B. exarata*.

The mismatch distribution analysis on the two COI haplogroup populations showed unimodal patterns, suggesting a history of expansion (Figures 3A,B). These results were supported with significantly negative values of *Fu’s Fs* and *Tajima’s D* (Supplementary Table S4). The expansion time estimated by τ from mismatch distribution analysis were 0.24 (CI: 0.21–0.28) Ma and 0.21 (CI: 0.15–0.26) Ma for the Haplogroups S and N, respectively (Supplementary Table S4). The BSP also demonstrated an increase in effective population sizes for the two haplogroups since a similar time as estimated by τ (Figures 3C,D). These estimated expansion times fall into the Pleistocene era.

## Discussion

In the present study, evidence from the mitochondrial COI gene and nine nuclear microsatellite loci was combined to reconstruct the phylogeographical history of *B. exarata*. Consistent with many previous studies, our data also suggested that the species remained the imprint of divergence in the two marginal seas around their range. Population expansion and a significant pattern of IBD were also detected.

### Phylogeographical Structure of *B. exarata*

The COI gene analysis unveiled a significant north-south differentiation in *B. exarata* populations. Nuclear microsatellite data also demonstrated a similar but weaker genetic structure, indicating a stronger gene flow that might be mediated by paternal line. Nonetheless, the phylogeographic structure pattern implies a historical divergence, and two origins in the north and south, respectively. The estimated *t_MRCA_* for the two COI haplogroups falls into Pleistocene era, during which the global climate changed drastically with the alternation of glaciation and deglaciation periods. The dramatic climate change had profound impacts on species distribution and abundance and played an important role in reconstructing their genetic structures (Dynesius and Jansson, 2000; Hewitt, 2000, 2004). In marine environments, historical glaciation is an effective force in generating intraspecific genetic splits, such as in the Indo-West Pacific (Kirkendale and Meyer, 2004), Atlantic and Mediterranean basins (Calderon et al., 2008; Cunha et al., 2008), as well as the Northwestern Atlantic region (Wares, 2002). During the Pleistocene glaciation, the sea level dropped 130–150 m in the northwest Pacific, and the shelf of the Bohai Sea, Yellow Sea, and East China Sea were mostly exposed, causing a large loss of habitat for *B. exarata*. In this study, the observed distribution pattern of the two haplogroup populations and estimated *t_MRCA_* for the two COI haplogroups imply that the ancestors of *B. exarata* were probably isolated and survived separately in the Sea of Japan and the Okinawa Trough, two marginal seas left during glaciation lower sea levels (Tamaki and Honza, 1994; Wang, 1999; Liu et al., 2006). A similar pattern was also observed for clam species (Kong and Li, 2009; Liu et al., 2011), marine fishes (Liu et al., 2007; Shen et al., 2011), and mitten crab (Xu et al., 2009) in this region, all of which suggested that the isolation in the marginal seas during the glaciation lower sea levels have shaped the patterns of phylogeographic distribution in the Northwestern Pacific.

On the other hand, the strong pattern of IBD in *B. exarata* populations is most likely attributable to the limited dispersal ability of 4–10 days of planktonic larval stage (You et al., 2003). Limited movement ability of adults and a short planktonic larval stage could promote and maintain the genetic differentiation in marine species (Hellberg, 1996; Shanks et al., 2003; Ye et al., 2015). For example, the clam *Coelomactra antiquate* showed highly significant genetic differentiation between populations from the Bohai Sea and East China Sea with a *F_ST_* value of 0.958 based on the COI gene. This species has a pelagic larval phase of around 9 days, a time duration that might be too short for the ocean currents to transfer the larvae a long distance and mix populations of with different genetic backgrounds (Wang, 2009). In contrast, there was no obvious phylogeographic structure detected in the mussel *Mytilus coruscus* along the coast of the East China Sea based on the COI gene, with *F_ST_* values from −0.036 to 0.097. This homogeneous phylogeographic structure could be explained by the same genetic background of the populations studied. The planktonic larvae stage of this species lasts up to 35 days, meaning that the ocean currents could promote the population mixture by facilitating the diffusion of planktonic larvae and generating the homogeneous phylogeographic structure of this species (Li et al., 2013).

### Historical Demography and Post-glaciation Expansion

All results obtained while investigating population demography support historical expansion of the two COI haplotype populations since the late Pleistocene era. The wide distributions of the two microsatellite groups could also explain the expansion from nuclear side. Interglaciation and post-glaciation expansion for marine species have been proven in many previous studies, and the expansion hypothesis is highly plausible in the Northwestern Pacific, which has many marginal seas (Cheang et al., 2012; Ni et al., 2012). When the sea level rose, the surviving populations of *B. exarata* in the two refugia might have rapidly colonized the new habitat available and experienced expansion history. Postglacial range expansions might have brought formerly isolated haplogroups into secondary contact at suture zones (Nielsen et al., 2004; Shen et al., 2011). Our study also detected an overlapping region of the two COI haplogroup populations from TJ to WH (Figure 1a), as well as shared genetic compositions of the two microsatellite groups across the entire study area (Figure 1b), indicating widespread secondary contact of *B. exarata* after expansion. When the sea level rose, the survived ancestors of northern populations may have expanded from the Sea of Japan through the Tsushima Strait and occupied the coastline of the Korea Peninsula westward. It the same time period, the ancestors of southern populations may have expanded out of the Okinawa Trough and colonized the coastline of the East China Sea.

## Conclusion

In our study, both the nuclear microsatellites and the mitochondrial COI gene revealed two genetic groups with clear geographic cline, as well as significant patterns of IBD in *B. exarata*. The estimated *t_MRCA_* for the two COI haplogroup populations falls into the Pleistocene era, providing evidence that the ancestors of *B. exarata* might have been isolated and diverged in the Sea of Japan and the Okinawa Trough as refugia during Pleistocene glaciation lower sea levels. Expansion of the two genetic groups since the late Pleistocene era may have occurred via recolonization of the recovered habitat when the sea level rose in deglaciation periods. The present genetic structure could have been promoted and maintained by limited mobility of adults and a short planktonic larval stage of *B. exarata*.

## Author Contributions

L-YS was responsible for the overall experiment and writing of this paper. JL and S-QW provided some help in sampling and experiment. JH provided financial support and worked on the experimental plan.

## Conflict of Interest Statement

The authors declare that the research was conducted in the absence of any commercial or financial relationships that could be construed as a potential conflict of interest.

## Footnotes

1 http://www.bio.sdsu.edu/pub/andy/IBD.html

2 http://taylor0.biology.ucla.edu/structureHarvester/
