# Supplementary material for: Historical Refugia and Isolation by Distance of the Mud Snail, Bullacta exarata (Philippi, 1849) in the Northwestern Pacific Ocean
Source: Front Genet. 2018 Oct 22;9:486. doi: 10.3389/fgene.2018.00486 (PMC6204408; doi:10.3389/fgene.2018.00486)
Supplement: Supplementary file 1 [file Table_1.DOCX]

**Table S1**

Sampling information and COI haplotype distribution of *B. exarata* populations.

| Population  ID | Sample  site | Sample  size | Date of  collection | Coordinate of  sample site | COI haplotype distribution |
| --- | --- | --- | --- | --- | --- |
| DD | Dandon | 32 | Aug. 2009 | 39°47′N, 123°59′E | Hap11(18), 12, 13(2), 14-24 |
| DL | Dalian | 32 | Aug. 2009 | 38°52′N, 121°35′E | Hap5(8), 11, 13(3), 25, 27-29, 30(4), 31-42 |
| JZ | Jinzhou | 31 | July 2009 | 40°52′N, 121°09′E | Hap5(5), 13(3), 29(3), 30(4), 63(2), 64(4), 65-67, 68(2), 69-73 |
| TJ | Tianjin | 34 | Aug. 2010 | 38°46′N, 117°44′E | Hap5(7), 13(7), 29(5), 30(3), 49(3), 50, 61, 64, 112(2), 113(2), 114, 115 |
| DY | Dongying | 30 | July 2010 | 37°26′N, 118°57′E | Hap5(9), 49(11), 54-63 |
| WH | Weihai | 30 | Sep. 2017 | 37°12′N, 122°38′E | Hap5(4), 13(3), 29(2), 30(4), 56, 63, 66, 68, 113, 116(2), 117-123, 124(2), 125 |
| QD | Qingdao | 34 | July 2010 | 36°03′N, 120°24′E | Hap44, 49(25), 105-107, 108(2), 109-111 |
| LYG | Lianyungang | 30 | Sep. 2013 | 34°45′N, 119°31′E | Hap49(17), 74, 75(2), 76-85 |
| DT | Yancheng | 30 | Sep. 2013 | 33°34′N, 120°37′E | Hap1, 3, 43(2), 44-48, 49(17), 50-53 |
| XT | Nantong | 30 | Sep. 2013 | 32°23′N, 121°08′E | Hap3, 48, 49(13), 136, 137(2), 138-149 |
| CMD | Shanghai | 30 | May 2013 | 31°36′N, 121°50′E | Hap2, 3(2), 4, 6-10, 26, 49(20) |
| NB | Ningbo | 30 | April 2014 | 29°42′N, 121°53′E | Hap3(2), 49(15), 50, 55, 86-96 |
| PY | Wenzhou | 29 | July 2013 | 27°35′N, 120°39′E | Hap43, 49(16), 50(2), 97, 98(2), 99-104 |
| XM | Xiamen | 30 | July 2013 | 32°03′N, 121°43′E | Hap3(2), 49(14), 89, 102(2), 108, 126-135 |

Note: Numbers in the parentheses are numbers of individuals with the corresponding haplotypes, and the number of haplotype is one if without following a parenthesis.

**Table S2**

Basic information of the nine microsatellite loci analyzed in this study

| Locus | Repeat Motif | Primer sequences (5’-3’) | Allele range (bp) | Ta (℃) | Reference |
| --- | --- | --- | --- | --- | --- |
| Bex 2 | (CA)17 | F: TTAACCCCGCACAAACATA  R: GCCCTTCTCAAACCTACCT | 275-287 | 61 | Du *et al.* 2010 |
| Bex 5 | (CA)37 | F: GAATACAGCCGTATGATGAC  R: CGGAAAGCAAGTGAAGAGG | 290-310 | 54 | Du *et al.* 2010 |
| Bex 6 | (TC)13 | F: AGTCGAATTTCCCTGTTATCC  R: AGTGCTGAGCGGGTTTATGG | 190-192 | 61 | Du *et al.* 2010 |
| Bex 7 | (CT)14 | F: CTACCAGCCAGTCTTTTC  R: ATGTACCCTCTCATAACCC | 224-226 | 56 | Du *et al.* 2010 |
| Bex 8 | (TC)24 | F: ATCTAGTGTTGTTGGTGAAATAC  R: GAGAGATAAAAATGGGGAGGT | 178-202 | 56 | Du *et al.* 2010 |
| Bex 9 | (GA)28 | F: AGGACAGGGAGGAAAAAGC  R: TAACAATACCGAGAGGTGAAC | 169-201 | 58 | Du *et al.* 2010 |
| Bex 12 | (TC)34 | F: CCAGAAACATTAGATGGCTCC  R: TACTAAGGCGTGGTTGAAAAG | 205-221 | 56 | Du *et al.* 2010 |
| Bex 15 | (GA)11 | F: AAGAGTGAGAAAGGGGAGC  R: ATGGTGTCTAAGAATAATGGG | 267-269 | 58 | Du *et al.* 2010 |
| Bex 28 | (AC)26 | F: ACATTGAGGGCATTCAGGAG  R: AGGATCAGAAGGCGACAGAA | 123-175 | 58 | Wang *et al.* 2010 |

**Table S3**

Pairwise *F_ST_* values based on nine microsatellites (above diagonal) and the mitochondrial COI gene (below diagonal)

|  | DD | DL | JZ | TJ | DY | WH | QD | LYG | DT | XT | CMD | NB | PY | XM |
| --- | --- | --- | --- | --- | --- | --- | --- | --- | --- | --- | --- | --- | --- | --- |
| DD | – | 0.020* | 0.025* | 0.031* | 0.117* | 0.033* | 0.120* | 0.187* | 0.125* | 0.133* | 0.157* | 0.143* | 0.011 | 0.131* |
| DL | 0.399* | – | 0.144* | 0.062* | 0.095 | 0.114* | 0.143* | 0.087* | 0.190* | 0.130* | 0.062* | 0.106* | 0.159* | 0.185* |
| JZ | 0.453* | 0.000 | – | 0.041* | 0.093* | 0.008 | 0.097* | 0.294* | 0.191* | 0.206* | 0.255* | 0.146* | 0.098* | 0.093* |
| TJ | 0.412* | 0.084* | 0.078* | – | 0.004 | 0.000 | 0.014 | 0.148* | 0.064* | 0.065* | 0.101* | 0.033* | 0.006 | 0.021* |
| DY | 0.581* | 0.372* | 0.373* | 0.149* | – | 0.008 | 0.003 | 0.123* | 0.028* | 0.025* | 0.086* | 0.018* | 0.020* | 0.009 |
| WH | 0.437* | 0.000 | 0.000 | 0.061* | 0.364* | – | 0.016 | 0.232* | 0.092* | 0.101* | 0.183* | 0.064* | 0.018* | 0.005 |
| QD | 0.892* | 0.812* | 0.821* | 0.625* | 0.351* | 0.832* | – | 0.177* | 0.024* | 0.056* | 0.147* | 0.005 | 0.008 | 0.000 |
| LYG | 0.886* | 0.802* | 0.811* | 0.610* | 0.335* | 0.821* | 0.009 | – | 0.154* | 0.084* | 0.008 | 0.075* | 0.197* | 0.227* |
| DT | 0.887* | 0.804* | 0.813* | 0.612* | 0.337* | 0.824* | 0.014 | 0.000 | – | 0.006 | 0.130* | 0.003 | 0.013 | 0.024* |
| XT | 0.846* | 0.757* | 0.764* | 0.563* | 0.282* | 0.766* | 0.019* | 0.008* | 0.002* | – | 0.056* | 0.023* | 0.000 | 0.064* |
| CMD | 0.880* | 0.795* | 0.804* | 0.601* | 0.322* | 0.813* | 0.029* | 0.009* | 0.000 | 0.000 | – | 0.068* | 0.148* | 0.173* |
| NB | 0.874* | 0.790* | 0.798* | 0.599* | 0.322* | 0.806* | 0.014* | 0.002 | 0.000 | 0.006* | 0.000 | – | 0.021* | 0.029* |
| PY | 0.861* | 0.774* | 0.782* | 0.581* | 0.303* | 0.787* | 0.016* | 0.003 | 0.003 | 0.016* | 0.020* | 0.015* | – | 0.000 |
| XM | 0.876* | 0.792* | 0.800* | 0.601* | 0.325* | 0.808* | 0.007 | 0.002 | 0.001 | 0.009* | 0.008* | 0.000 | 0.000 | – |

Note: ^*^The *F_ST_* values are significant after the sequential Bonferroni correction. The negative values exported by software were corrected to zero.

**Table S4**

Neutral test and historical demography parameters for two groups of *B. exarata* populations based on the COI gene.

|  | Neutrality | | Mismatch distribution analysis | | |
| --- | --- | --- | --- | --- | --- |
|  | Tajima’s *D* | Fu’s *Fs* | Tau (τ) | Theta 0 (*θ_0_*) | Theta 1 (*θ_1_*) |
| Haplogroup N | -1.83625** | -25.14867** | 3.348 (2.354-4.076) | 3.357 | 99999 |
| Haplogroup S | -2.66183 | -25.99067** | 3.740 (3.209-4.451) | 0.000 | 99999 |

Note: τ, reflects the location of the mismatch distribution crest, provides a rough estimate of the time when rapid population expansion started. *θ_0_* and *θ_1_*, compound parameter representing the mutation rate and the female effective population size before and after expansion, respectively.

**Table S5**

The information of all reagents and platforms used in this article.

| Reagent and platforms | Manufacturer |
| --- | --- |
| Hexadecyl trimethyl ammonium bromide | Amresco, America |
| Polyvinyl pyrrolidone | Amresco, America |
| Tris | Amresco, America |
| Ethylenediaminetetraacetic acid | Amresco, America |
| Proteinase K | Takara, Japan |
| SYBR Safe DNA Gel Stain | Invitrogen, America |
| Agarose | Biowest, France |
| 2×EasyTaq PCR SuperMix | TransGen Biotech, China |
| 1×TE | TransGen Biotech, China |
| Trans2K DNA Marker | TransGen Biotech, China |
| Trans15K DNA Marker | TransGen Biotech, China |
| Thioglycol | TransGen Biotech, China |
| Ethyl alcohol absolute | TransGen Biotech, China |
| Hydrochloric acid | TransGen Biotech, China |
| Sodium hydroxide | TransGen Biotech, China |
| AceticAcid | TransGen Biotech, China |
| Sodium chloride | TransGen Biotech, China |
| Sodium dodecyl sulfate | TransGen Biotech, China |
| Trichloromethane | TransGen Biotech, China |
| Isoamyl alcohol | TransGen Biotech, China |
| Microscope | Aote optical instrument co. LTD, China |
| Stereoscopic microscope | Zeiss, Germany |
| Pure water purification system | Millipore, America |
| Refrigerated centrifuge | Eppendorf, Germany |
| Pipettor | Eppendorf, Germany |
| PCR Thermocycle Instrument | Applied Biosystems, America |
| Electrophoresis apparatus | BioRad, America |
| Enzyme-labeled instrument | BioTek, America |
| - Constant temperature cycle water bath | PolyScience, America |
| [Autoclave](javascript:;) | Sanyo, Japan |


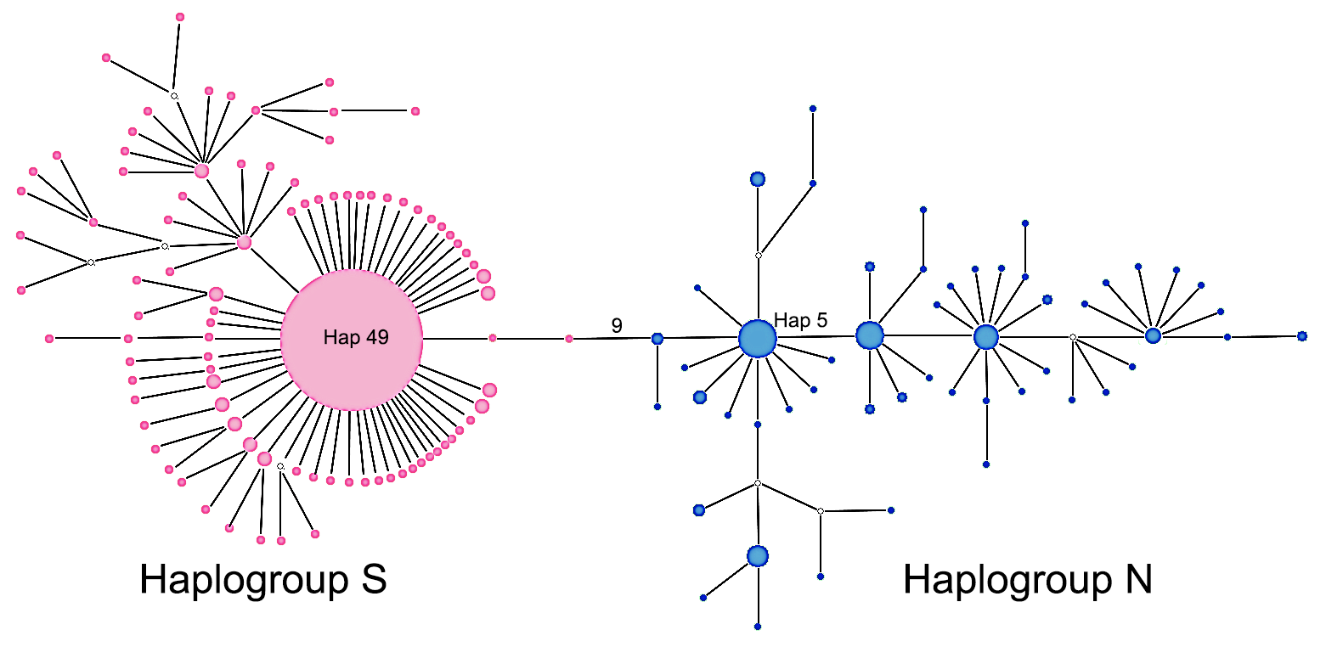


**Figure S1. The MJ network of 149 COI haplotypes.**

White circles represent median vectors, which may be un-sampled or extinct haplotypes. Circle sizes are proportional to haplotype frequency, and the smallest circle represents one haplotype. Each branch represents one mutational difference unless labelled with number indicating the number of mutations.


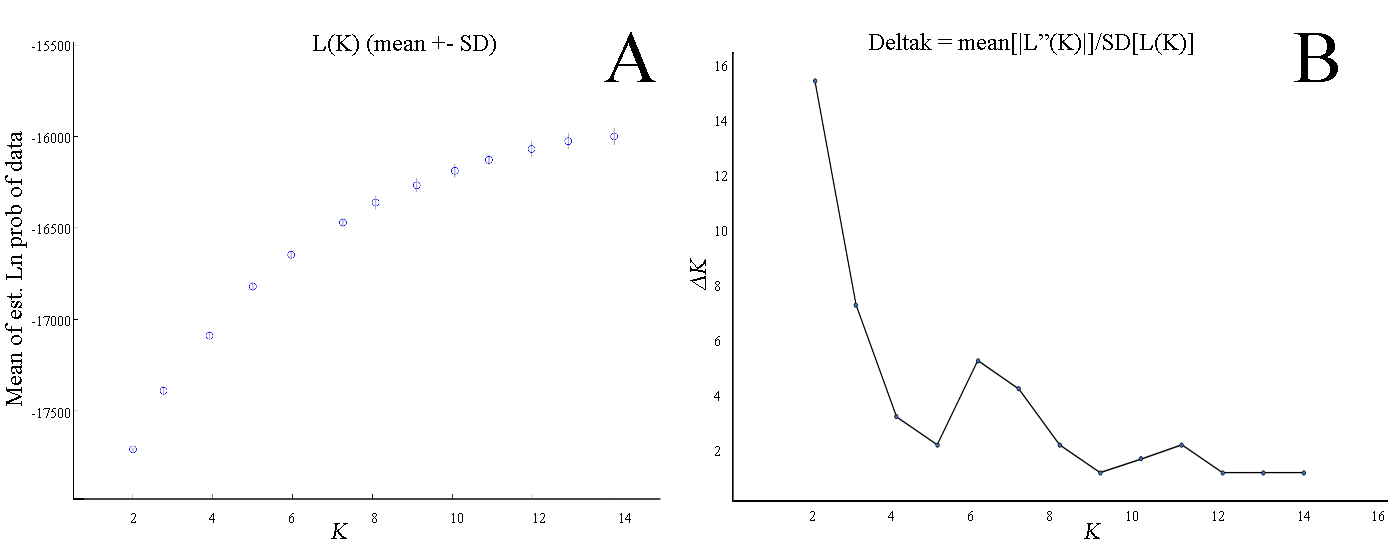


**Figure S2.** (A) The log-likelihood score [lnPr(*D*)] and (B) the rate of change in the log probability of data between successive *K* values (*ΔK*).
